# Relationship between postpartum psychological disorders and emotion regulation strategies: A cross-sectional study

**DOI:** 10.1097/MD.0000000000038294

**Published:** 2024-05-31

**Authors:** Rui Li, Meng-Yao Liang, Yue Wu, Xiao-feng Zheng, Lu Ma, Hong Song

**Affiliations:** a Department of Nursing, The Second Affiliated Hospital of Xuzhou Medical University, Xuzhou, Jiangsu, China; b Department of Nursing, The Sixth People’s Hospital of Nantong, Nantong, Jiangsu, China.

**Keywords:** correlation, emotional regulation, postpartum women, psychological disorders

## Abstract

To explore the relationship between postpartum psychological disorders and emotion regulation strategies and analyze the influencing factors of postpartum psychological disorders. This study was conducted using a cross-sectional design. A total of 230 postpartum women hospitalized in the Second Affiliated Hospital of Xuzhou Medical University from October 2022 to March 2023 were selected as the investigation objects. A general data questionnaire, Hamilton Anxiety Scale, Hamilton Depression Scale, Pittsburgh Sleep Quality Index, and Emotion Regulation Questionnaire were administered to the enrolled women. Pearson correlation analysis was used to assess the association between the Hamilton Anxiety Scale, Hamilton Depression Scale, Pittsburgh Sleep Quality Index, and Emotion Regulation Questionnaire. Furthermore, logistic regression was employed to assess the influencing factors of postpartum psychological disorders. Pearson correlation analysis showed that cognitive reappraisal was negatively correlated and expression inhibition was positively associated with anxiety, depression, and sleep quality symptoms (all *P* < .05). Logistic regression results demonstrated that the mode of delivery, number of births, feeding method, and pressure to breastfeed were risk factors affecting postpartum psychological disorders (*P* < .05). Cognitive reappraisal is an effective emotion regulation strategy that can relieve postpartum psycho-neurological symptoms by reducing the symptoms of anxiety, depression, and sleep disorders. Along with encouraging pregnant women to adopt positive emotional regulation strategies, medical personnel should focus on the stress associated with cesarean section, artificial feeding, and pressure to breastfeed and adopt required intervention measures to decrease the occurrence of postpartum psychological and neurological symptoms.

**Key Points**

The incidence of postpartum mental and psychological symptoms in parturients is high. In addition to encouraging parturients to take positive emotional regulation strategies, medical staff should pay more attention to primiparas with cesarean section, non-breastfeeding and breastfeeding pressure, and adopt intervention measures when necessary to reduce the occurrence of postpartum mental and psychological symptoms.

## 1. Introduction

China’s birth policy has moved toward a new stage following the implementation of the “single two-child” policy in 2013 and the “comprehensive two-child” in 2016.^[1]^ Although China’s birth population has exhibited a gradual upward trend after the execution of the new policy, it still fails to meet the fertility expectations of the Chinese population. The total fertility rate of the population is approximately 1.7, indicating that the problem of low birth population and fertility rate still exists. In 2021, China attempted to alleviate the population pressure by reducing the aging population via the enactment of the policy of “a couple can have three children” and supporting measures. Additionally, the fertility policy was further optimized by improving the maternity leave and insurance system as well as strengthening the supporting roles of taxation and housing.^[2]^ Consequently, research on the fertility intention of “three children” initiated another wave of hot debate among scholars.^[2]^ In response to the new fertility policy, women of childbearing age may experience more uncertainty, higher psychological pressure, and decreased happiness due to exposure to multiple pressures, making them prone to encounter female psychological problems. Moreover, the sharp decline in various hormone levels after delivery may cause women to develop persistent depression, which can manifest as a lack of interest and slow thinking.^[2]^ Apart from the above changes, the stress of identity transition and appearance anxiety related to body management and hair loss can further escalate postpartum psychological disorders.^[3]^ The term postpartum psychological disorders refers to postpartum mental and behavioral disorders and abnormal psychological states, primarily manifesting as postpartum anxiety, depression, and sleep disorders.^[4]^ Previous studies have shown that the incidence of postpartum anxiety and depression ranges from 15% to 85%,^[5]^ whereas sleep disorders can reach an occurrence rate as high as 80%.^[6]^ Postpartum anxiety and depression can not only affect the physical and mental health of parturients but also influence the normal growth and development of their newborns and exert various adverse effects on their family relations and social relationships.^[7]^ Furthermore, maternal sleep quality affects the mother as well as has a considerable impact on the newborn infant, with good maternal sleep quality reported to support the positive and healthy development of infants.^[8]^ Prior research has suggested that maternal sleep quality is positively correlated with the development of infant behavior within 4 months postpartum.^[9]^ However, other studies have indicated that less than one-third of postpartum women seek treatment for mental and psychological problems, of which only an extremely small proportion receive mental healthcare.^[10]^ The untreated postpartum psychological disorders can in turn lead to developmental delays in the children and impair the mother-child connection.^[11]^ These problems can eventually damage the physical and mental health of the affected mothers and infants, potentially resulting in maternal self-injury and suicide and eventually placing a heavy burden on their families and society.^[12]^ Therefore, understanding the influencing factors of postpartum psychological disorders and actively modifying the controllable factors is critical in reducing the occurrence of postpartum psychological disorders as well as in improving the mental quality of life and physical and mental health of the mothers.

Emotion regulation strategies refer to the measures individuals undertake to cope with negative emotions, encompassing processes that enable them to influence the occurrence, experience, and expression of their emotions.^[13]^ Cognitive reappraisal and expression inhibition are the most common and effective antecedent- and response-focused strategies, respectively. Cognitive reappraisal involves individuals changing their understanding of emotional events to reduce negative emotions and increase the expression of positive emotions, thereby alleviating negative emotions. Expression inhibition entails individuals suppressing persistent emotional expression behaviors, including negative and positive emotions.^[14]^ Emotion regulation strategies are closely related to the incidence of psychological disorders.^[15]^ Therefore, deciphering the correlation between emotion regulation strategies and postpartum psychological disorders and encouraging women to adopt effective regulation strategies will contribute to reducing the occurrence of such psychological disorders.

In this study, we conducted a questionnaire survey of 230 postpartum participants to investigate the occurrence status of their psychological disorders, explore the correlation between their psychological disorders and emotion regulation strategies, and analyze the influencing factors of their psychological disorders. Furthermore, this study hoped to guide clinical staff to actively implement interventions and provide a scientific basis for reducing the incidence of postpartum psychological disorders.

## 2. Methods

### 2.1. Participants

Postpartum women hospitalized for reexamination in the Second Affiliated Hospital of Xuzhou Medical University from October 2022 to March 2023 were selected for this study using the convenience sampling method. The participant inclusion criteria were as follows: a pregnancy of 37 to 42 weeks, full-term delivery, and single fetus, with newborn in good condition (e.g., no organic diseases); age >18 years; no history of mental illness, intellectual disabilities, personality disorders, anxiety, depression, or other mental symptoms before delivery; and maternal informed consent and voluntary participation. Participants were excluded if they met any of the following criteria: a history of alcoholism or alcohol/controlled substance abuse or currently undergoing psychological treatment.

#### 2.1.1. Sample size calculation

The formula used for calculating the minimum sample size of the cross-sectional survey is as follows: $n=\frac{\mu_{\alpha /2}^{2}\pi \left(1-\pi \right)}{\sigma^{2}}$, where bilateral α = 0.1; therefore, μ_α/2_ = 1.64. Additionally, the reported incidence of postpartum mental disorders in Chinese women was 21.4%^[16]^; thus, π = 0.214. Lastly, σ (allowable error) = 0.05 in this study. Hence, the minimum sample size was 182. Considering 20% of sample shedding, the minimum sample size was ultimately selected as 228.

### 2.2. Research tools

#### 2.2.1. General data collection

Demographic characteristics included age, marital status, mode of delivery, number of births, feeding method, pressure to breastfeed, place of residence, educational level, occupation, and per capita monthly income of the family.

#### 2.2.2. Hamilton Anxiety Scale

This scale was originally developed by Hamilton while its Chinese version was translated by Tang et al.^[17]^ The Hamilton Anxiety Scale (HAMA) contains 14 items, which are divided into 2 major factors: somatic anxiety and psychic anxiety. All items are scored on a 4-level scale ranging from 0 (none) to 4 (severe), wherein a score of 29 denotes severe anxiety; ≥21, obvious anxiety; ≥14, certain anxiety; and ≥7, potential anxiety. In this study, 14 points was the critical value, with a HAMA score of >14 suggesting anxiety. It is convenient and practical in clinic, and can better identify anxious people. Lastly, the Cronbach’s α coefficient of this scale was 0.900.

#### 2.2.3. Hamilton Depression Scale

This scale was initially developed by Hamilton and subsequently revised by Tang et al.^[18]^ The Hamilton Depression Scale (HAMD) comprises 24 items, which are scored via a 5-level scoring method (i.e., 0 [none] to 4 [extremely severe]), while a few items utilize a 3-level scoring method (i.e., 0 [none] to 2 [severe]). Moreover, HAMD scores ≥20 signify depression, where a score of 20 to 35 indicates mild to moderate depression, and a score of >35 demonstrates severe depression. In this study, a total HAMD score of 20 was set as the cutoff value; therefore, HAMD scores ≥20 indicated depression. The total score of HAMD scale can better reflect the severity of the disease, and it is convenient and practical in clinic, and can better identify depressed people. Finally, the Cronbach’s α coefficient of this scale was 0.850.

#### 2.2.4. Pittsburgh Sleep Quality Index

This scale has 18 self-rated items, encompassing 7 components: sleep quality, fall asleep time, sleep duration, sleep efficiency, sleep disorders, hypnotic drugs, and daytime dysfunction.^[19]^ Each component is scored from 0 (never) to 3 (always), with the total score ranging from 0 to 21. In the Pittsburgh Sleep Quality Index (PSQI), higher scores imply poorer sleep quality, with a total score of >7 suggesting poor sleep quality. Lastly, the Cronbach’s α coefficient of this scale was 0.920.

#### 2.2.5. Emotion Regulation Questionnaire

This questionnaire was developed and validated by Preece et al.^[20]^ The Emotion Regulation Questionnaire (ERQ) is divided into 2 dimensions: cognitive reappraisal (6 items) and expression inhibition (4 items). The ERQ items are scored on a 7-point scale ranging from 1 (extremely inconsistent) to 7 (extremely consistent). Higher scores on either dimension indicate a higher tendency of the individual to use the corresponding emotion regulation strategy, with the frequent employment of one strategy not affecting the utilization of the other strategy. Finally, the Cronbach’s α coefficients of the 2 dimensions of cognitive reappraisal and expression inhibition were 0.940 and 0.830, respectively.

In the text introduction, a HAMA score >14, (or) a HAMD score ≥20, (or) a PSQI score >7 are considered to have psychiatric symptoms. If all 3 items do not match, it is “0,” otherwise it is “1.”

### 2.3. Data collection and quality control

The general information questionnaire and PSQI questionnaires were distributed and collected by the nurses during the 42-day postpartum review. The women voluntarily participated in this study, and they accurately filled in the questionnaires. The HAMA and HAMD scales are reviewed jointly by 2 trained raters. The method of conversation and observation is generally adopted. After the inspection, 2 reviewers scored independently. After the completed questionnaire was collected, 1 researcher from our research group who was trained and qualified in data collection methods inputted the data into Excel, while another researcher verified the data to ensure accurate data entry. Furthermore, the questionnaire was distributed and collected in a separate conference room to ensure the privacy of the participants, and a small token of appreciation was provided to the participants after they completed the questionnaire. The questionnaire required approximately 15 to 20 minutes to be completed.

### 2.4. Ethical considerations

Although this study was conducted anonymously, this research did not involve unethical practices or human clinical trials. Moreover, we ensured that this study had no adverse effects on the participants’ physical and mental health, and we undertook stringent measures to address all ethical considerations. First, we obtained ethical approval from the hospital before issuing the questionnaire to the participants. Second, we comprehensively explained the purpose and significance of the study to the prospective participants and recruited only those who provided informed consent. Third, we ensured that the participants’ responses were only used for this study and gave the participants the choice to stop answering the questionnaires at any time. Finally, this study was approved by Ethics Committee of the Second Affiliated Hospital of Xuzhou Medical University (No: zy2022090013).

### 2.5. Statistical analysis

Data from this study were checked by 2 researchers and entered into Excel 2016. All statistical analyses were performed using SPSS24.0 software (IBM Corp, Armonk, NY). Count data were represented by examples and percentage (%), and the Chi-square test was used for analysis. Measurement data were expressed as mean ± standard deviation (*X* ± *S*). Pearson correlation and logistic regression analyses were conducted to assess the influencing factors. The statistical significant level was set at α = 0.05.

## 3. Results

### 3.1. Demographic characteristics of the participants

**Figure 1.**
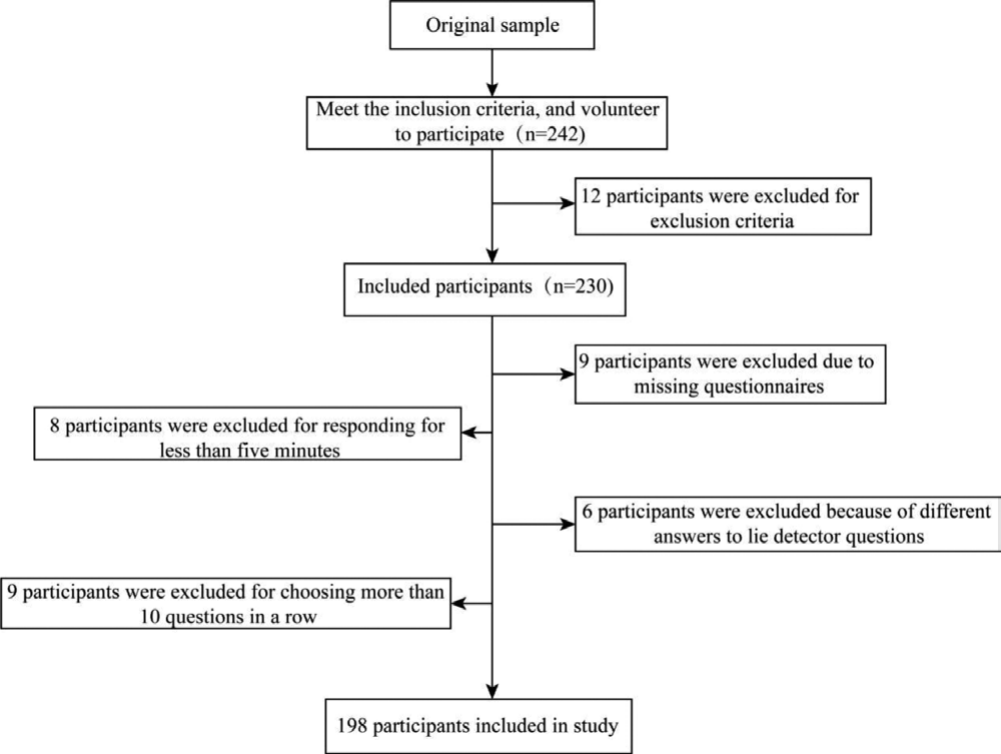
Flowchart.

A total of 230 pregnant women were included in this study. Of them, 32 were excluded from the study for reasons including ≥20% of missing items in the questionnaire, questionnaire response time <5 minutes, and the same response option selected for more than 10 consecutive items. A total of 198 valid questionnaires were finally collected, leading to an effective recovery rate of 86.09% (Fig. 1). The 198 parturients had an age range of 23 to 45 years and an average age of 29.36 ± 5.33 years. The demographic data of all included participants are shown in Table 1.

**Table 1:** General demographic data of the enrolled participants (n = 198).

Variable	Frequency	%
Age (yr)		
<30	97	48.99
30–40	73	36.87
>40	28	14.14
Marital status		
Married	195	98.48
Spinsterhood	2	1.01
Divorced	1	0.51
Delivery mode		
Vaginal delivery	152	76.77
Cesarean delivery	46	23.23
Residence		
Urban	94	47.47
Cities and towns	68	34.34
Rural	36	18.19
Monthly income (RMB)		
<3000	29	14.65
3000–5000	46	23.23
5001–7000	62	31.31
7001–10,000	33	16.67
>10,000	28	14.14
Degree of education		
Primary education	40	20.20
Secondary education	56	28.28
Junior college	49	24.75
Bachelor degree or above	53	26.77
Occupation		
Permanent	122	61.62
Freelance	33	16.67
No occupation	43	21.72

### 3.2. Scores of the enrolled participants on all questionnaires

The responses of the participants to all questionnaires are scored, analyzed, and summarized in Table 2. In this study, 86 of the total 198 participants exhibited postpartum psychological disorders, resulting in an incidence of 43.43%. Among them, 48 participants demonstrated postpartum anxiety, depression, and sleep disorders, leading to an occurrence rate of 24.24%, consistent with the findings of Lawson et al.^[21]^ However, the postpartum anxiety incidence of 28.79% in our study differed from that of 10.0% to 20.0% reported by Pawluski et al^[22]^ and 40.91% in the study by Li et al.^[23]^ Similarly, the postpartum depression incidence of 37.37% was much higher than that of 13.0% to 19.7% detected in previous studies.^[24–26]^ In this study, the incidence of 26.77% of postpartum sleep disorders was slightly higher than that of 24.0% reported by Gessesse et al.^[27]^ Another study by Okun et al^[28]^ highlighted that sleep conditions are closely related to anxiety and depression and that sleep disorders can aggravate postpartum anxiety and depression.

**Table 2:** Postpartum psychological disorders and emotion regulation strategies according to the various questionnaire scores (n = 198).

	Min	Max	Mean	Standard deviation
HAMA	1	42	11.33	3.12
HAMD	2	46	16.27	5.43
PSQI	0	21	5.82	1.34
ERQ:Cognitive reappraisal	6	42	29.48	5.67
ERQ:Expressive inhibition	4	28	19.33	4.11

ERQ = Emotion Regulation Questionnaire, HAMA *=* Hamilton Anxiety Scale, HAMD = Hamilton Depression Scale, PSQI = Pittsburgh Sleep Quality Index.

### 3.3. Correlation analysis of postpartum psychological disorders and emotion regulation strategies

Pearson correlation analysis was used to assess the correlation between postpartum psychological disorders and the emotion regulation strategies of the participants. The results showed that cognitive reappraisal was negatively correlated and expression inhibition was positively correlated with anxiety, depression, and sleep quality symptoms (all *P* < .05). The specific correlation coefficients are listed in Table 3.

**Table 3:** Correlation between postpartum psychological disorders and emotion regulation strategies according to *r* value.

Variable	Cognitive reappraisal	Expressive inhibition	HAMA	Somatic anxiety	Psychic anxiety	HAMD	PSQI
ERQ:Cognitive reappraisal	1						
ERQ:Expressive inhibition	−0.642**	1					
HAMA	−0.671**	0.464**	1				
Somatic anxiety	−0.432**	0.372*	0.683**	1			
Psychic anxiety	−0.522**	0.420**	0.640**	0.558**	1		
HAMD	−0.504*	0.527*	0.592*	0.610*	0.683**	1	
PSQI	−0.523*	0.611*	0.623*	0.653*	0.806*	0.657*	1

ERQ = Emotion Regulation Questionnaire, HAMA *=* Hamilton Anxiety Scale, HAMD = Hamilton Depression Scale, PSQI = Pittsburgh Sleep Quality Index.

* *P* < .05,

** *P* < .01 (2-tailed).

### 3.4. Analysis of the influencing factors of postpartum psychological disorders

The general data of the participants with psychological disorders are analyzed and summarized in Table 4. The results showed that the mode of delivery, number of births, feeding method, and pressure to breastfeed were the risk factors associated with postpartum psychological disorders (*P* < .05).

**Table 4:** Analysis of the influencing factors of postpartum psychological disorders (n = 198).

Predictive variable	Psychological disorders group (n* *= 86)	Normal group (n* *= 112)	*χ^2^*	*P*
Age (yr)			1.287	.525
<30	38	48		
30–40	33	50		
>40	15	14		
Marital status			1.348	.510
Married	84	111		
Spinsterhood	1	1		
Divorced	1	0		
Delivery mode			4.177	.041
Vaginal delivery	60	92		
Cesarean delivery	26	20		
Number of births			9.840	.007
1	66	67		
2	19	33		
≥3	1	12		
Feeding method			8.023	.018
Breastfeeding	28	59		
Mixed feeding	37	33		
Artificial feeding	21	20		
Pressure to breastfeed			7.066	.008
Yes	54	49		
No	32	63		
Residence			2.682	.262
Urban	39	55		
Cities and towns	27	41		
Rural	20	16		
Degree of education			5.027	.170
Primary education	15	25		
Secondary education	22	34		
Junior college	28	21		
Bachelor degree or above	21	32		
Occupation			3.433	.180
Permanent	49	73		
Freelance	13	20		
No occupation	24	19		
Monthly income (RMB)			8.287	.082
<3000	10	19		
3000–5000	15	31		
5001–7000	33	29		
7001–10,000	19	17		
>10,000	9	19		

### 3.5. Logistic regression analysis of the influencing factors of postpartum psychological disorders

The statistically significant factors in Table 4 were used as independent variables (values assigned in Table 5), whereas the occurrence of postpartum psychological disorders was considered the dependent variable (no = 0, yes = 1). Multivariate logistic regression analysis was then performed (α input = 0.05, α output = 0.10). The results of the specific regression analyses are presented in Table 6.

**Table 5:** Independent variable assignment for the logistic regression analysis of the influencing factors of postpartum psychological disorders.

Independent variable	Assigned value
Delivery mode	Vaginal delivery = 0, cesarean delivery = 1
Number of births	First birth (1) = 1, second birth (2) = 2, third birth or more (≥3) = 3
Feeding method	Breastfeeding = 1, mixed feeding = 2, artificial feeding = 3
Pressure to breastfeed	No = 0, yes = 1

**Table 6:** Logistic regression analysis of the influencing factors of postpartum psychological disorders (n = 198).

Variables	*B*	SE	Walds *χ^2^*	*P*	OR	95% CI
Constant	2.833	0.816	12.054	<.001	4.322	-
Delivery mode	1.335	0.622	4.607	.033	1.238	1.023–1.542
Number of births	−1.345	0.521	6.665	.006	0.810	0.634–0.938
Feeding method	1.006	0.484	4.320	.038	1.462	1.118–1.832
Pressure to breastfeed	0.552	0.185	8.903	<.001	1.543	1.201–1.838

Cl = confidence interval, OR = odds ratio, SE = standard error.

## 4. Discussion

The incidence of postpartum psychological disorders has been increasing in recent years. The term postpartum psychological disorders refers to postpartum mental and behavioral disorders and abnormal psychological states, primarily manifesting as postpartum anxiety, depression, and sleep disorders.^[4]^ Postpartum depression remains one of the most common postpartum psychological disorders, with sustained postpartum anxiety and depression seriously affecting the physical and mental health of mothers and newborns.^[7]^ There are some differences between the measurement results of this study and those of previous studies. This discrepancy in the incidence of postpartum psychological disorders could be attributed to the heterogeneity of the women included in the prior studies,^[22,23]^ as well as the method of delivery and measurement tools. Moreover, some women may show a certain degree of postpartum depression symptoms; however, they may not meet the definition of postpartum depression, resulting in a relatively higher incidence of postpartum depression symptoms in our study. Other studies have also suggested that poor sleep quality during prenatal pregnancy can cause postpartum anxiety and depression symptoms.^[29]^ All these findings indicate that the active detection and management of the sleep quality of pregnant women by clinical staff can serve as a preventive strategy against postpartum anxiety and depression.

Emotional regulation strategies have also been found to be closely related to postpartum psychological disorders. McRae and Gross^[30]^ showed that cognitive reappraisal was an effective way to relieve psychological distress and adapt to the surrounding environment. Correspondingly, an investigation by Shu et al^[31]^ confirmed that cognitive reappraisal can alleviate patients’ negative emotions, such as anxiety and sadness. In the current study, Pearson correlation analysis revealed that cognitive reappraisal was negatively correlated and expression inhibition was positively correlated with postpartum psychological disorders. This finding could be explained by the gradual change in a woman’s attitude toward events during postpartum maternity, characterized by the increasing expression of positive emotions. This change can cause a transition from negative emotional output to positive emotional output, which in turn can reduce negative emotions as well as relieve anxiety and depression and other conditions. The amelioration of anxiety and depression symptoms can further affect the sleep quality of the women, resulting in improved sleep quality. In contrast, expression inhibition (i.e., the continuous inhibition of the expression of emotions) after delivery may not be conducive to developing healthy regulation of emotions.^[32]^ These observations suggest that clinical medical staff should prioritize training postpartum women in their cognitive reappraisal ability, as well as effectively guiding them in understanding their problems across various perspectives and multiple thinking. Correct negative emotions and behaviors by changing individual cognitive processes and perceptions. Postpartum women need to learn to identify the influence of unreasonable thinking on emotions, and strengthen their own control of behavior and perception of pleasant emotions. Additionally, postpartum women should be encouraged to express their emotions, particularly maternal negative emotions.

Postpartum psychological disorders are associated with numerous factors. In this study, we found that primiparas who had undergone cesarean section, were non-breastfeeding, and reported pressure to breastfeed were more likely to experience psychological disorders. A study by Shelton and Cormier^[33]^ established that women who delivered by cesarean section had a higher likelihood of developing postpartum depression and sleep disorders. The reason for these observations may be that the pain arising from the surgical incision for cesarean section reduces the postpartum comfort of the participants, as well as decreases their bed rest and self-care ability. These changes make it challenging to quickly adapt to the transformed role of the postpartum caregiver, thereby escalating the probability of forming maternal negative emotions. Furthermore, Weldu et al^[34]^ suggested that infant feeding practice might be an influential factor in postpartum depression. Breastfeeding can strengthen the intimate relationship between a mother and her infant, and establishing this intimate relationship is crucial for postpartum happiness among mothers. The resulting increase in postpartum happiness can further reduce the chance of postpartum depression.^[35]^ A study by Chen et al^[36]^ also demonstrated that the increased pressure to breastfeed was a cause of postpartum psychological disorders. Considering that excessive psychological pressure is a known factor that leads to sleep disorders and even insomnia, the pressure to breastfeed may diminish maternal sleep quality, which in turn could induce postpartum anxiety and depression. Another research by Nakamura et al^[37]^ also showed that primiparas were more likely to experience postpartum anxiety and depression. Compared with menparas, primiparas may be relatively younger and have no birth experience, which can increase their psychological stress and eventually lead to the development of psychological disorders.

There are some limitations to this study. The questionnaire was collected in only one center, which may lead to bias and limit the universality of the study. Moreover, the small sample size and high loss rate reduce the validity of statistics to some extent.

## 5. Clinical significance and prospect

Clinical staff should focus on the management of postpartum psychological disorders via effective evaluation, prevention, and treatment measures, including actively assessing for postpartum psychological disorders, conducting timely psychological counseling of women at high risk for psychological disorders, and providing appropriate drug treatment and control if required. Additionally, efficiently using the Internet and nursing service platforms for disseminating puerperal health information education can facilitate postpartum women to smoothly cope with multiple pressures, such as hormone decline and role transformation. Moreover, women with anxiety, depression, and sleep disorders should undergo active treatment that takes into account their physical and psychological health to minimize the incidence of adverse events caused by postpartum psychological disorders.

Postpartum psychological disorders affect the physical and mental health of mothers and, if untreated, can lead to developmental delays in children and damage the mother-child relationship. In the follow-up, this study will carry out a longitudinal investigation to explore the correlation between postpartum psychological disorders and mother–child relationship, and dynamically understand the changing trend of postpartum psychological disorders and mother–child relationship.

## 6. Conclusion

Cognitive reappraisal is an effective emotion regulation strategy to relieve postpartum psychological disorders, particularly the symptoms of anxiety, depression, and sleep disorders. The mode of delivery, number of births, feeding method, and pressure to breastfeed were the influencing factors of postpartum psychological disorders. Along with encouraging women to adopt positive emotional regulation strategies, medical personnel should pay attention to primiparas who underwent cesarean section, were non-breastfeeding, and reported pressure to breastfeed and should implement the relevant intervention measures to mitigate the occurrence of postpartum psychological disorders. This study has a few limitations that should be considered. The questionnaires in this study were collected from only a single center, and the study had a small sample size with a high sample-shedding rate. Moreover, this study did not currently conduct qualitative interviews on postpartum clinical parturients due to the shortage of personnel and time. Therefore, based on the theory of mixed research methods, future studies should conduct quantitative research combined with semi-structured interviews to further explore postpartum mental health problems.

## Acknowledgments

We thank all participants in our study.

## Author contributions

**Conceptualization:** Rui Li, Meng-Yao Liang, Yue Wu, Xiao-feng Zheng, Lu Ma, Hong Song.

**Data curation:** Rui Li, Meng-Yao Liang, Yue Wu, Xiao-feng Zheng, Hong Song.

**Formal analysis:** Rui Li, Meng-Yao Liang, Yue Wu, Xiao-feng Zheng, Lu Ma, Hong Song.

**Funding acquisition:** Rui Li, Meng-Yao Liang, Yue Wu, Xiao-feng Zheng, Lu Ma, Hong Song.

**Investigation:** Rui Li, Meng-Yao Liang, Yue Wu, Xiao-feng Zheng, Lu Ma, Hong Song.

**Methodology:** Rui Li, Meng-Yao Liang, Yue Wu, Xiao-feng Zheng, Hong Song.

**Writing – original draft:** Rui Li.

**Writing – review & editing:** Rui Li, Lu Ma.

**Project administration:** Meng-Yao Liang, Yue Wu, Xiao-feng Zheng, Hong Song.

**Resources:** Lu Ma, Hong Song.

**Supervision:** Lu Ma.
